# World AIDS Day — December 1, 2014

**Published:** 2014-11-28

World AIDS Day draws attention to the current status of the human immunodeficiency virus/acquired immunodeficiency syndrome (HIV/AIDS) epidemic worldwide. The theme for this year’s observance on December 1 is “Focus, Partner, Achieve: An AIDS-Free Generation.”

The first cases of AIDS were reported more than 30 years ago in the June 5, 1981 issue of *MMWR*. Today, an estimated 35 million persons are living with HIV infection (1). Although AIDS-related deaths have fallen by 35% since 2005, an estimated 1.5 million persons died from AIDS in 2013 (1).

Global efforts, including the President’s Emergency Plan for AIDS Relief (in which CDC is a principal agency), have resulted in approximately 11.7 million persons in low-income and middle-income countries receiving antiretroviral therapy for HIV infection in 2013 (1). This is nearly 2 million more persons than in 2012 (1).

In the United States, nearly 648,500 persons diagnosed with AIDS have died since the first cases were reported (2), and approximately 50,000 persons become infected with HIV each year (3). An estimated 1.2 million persons in the United States are living with HIV infection (4).
